# Effect of a Combined Bleaching Regimen on the Microhardness of a Sealed Methacrylate-based and a Silorane-based Composite

**Published:** 2013-09

**Authors:** F Shafiei, S Doustfatemeh

**Affiliations:** a Dept. of Operative Dentistry, Biomaterial Research Center, School of Dentistry, Shiraz University of Medical Science, Shiraz, Iran.; b Undergraduate Student, School of Dentistry, Shiraz University of Medical Science, Shiraz, Iran.

**Keywords:** Bleaching, Microhardness, Sealant, Methacrylate-based composite, Silorane-based Composites

## Abstract

**Statement of Problem:** The use of tooth bleaching agents has been very popular treatment in dentistry. The bleaching agents have an inherent potential to impair surface properties of existing composite resin restorations.

**Purpose:** This study evaluated the effect of a combined bleaching regimen on the surface microhardness of a Silorane-based and a sealed methacrylate-based composite.

**Materials and Method:** Forty-five specimens of methacrylate-based composite (Ice) and 18 specimens of Silorane composite (Filtek Silorane, 3M ESPE; USA) were prepared and randomly divided into 5 (1-5) and 2 (6-7) groups (n=9), respectively. After 8-week aging, groups 1 and 6 were remained with no treatment. In groups 2, 4 and 5, the specimens were covered by a surface sealant and light cured. In groups 3, 4, 5 and 7, the specimens were bleached with hydrogen peroxide 40% and then carbamide peroxide 20% for seven days. In group 5, after bleaching, the sealant was removed by polishing. Surface microhardness was measured and the data were analyzed using one-way ANOVA and Tukey tests (α=0.05)*. *

**Results:** The microhardness values of groups 2 to 4 were significantly lower than that of group1 (*p *<0.05). There was no significant difference among groups 1, 5, 6 and 7 (*p*> 0.05).

**Conclusion:** The combined bleaching regimen used in this study had a substantial negative effect on methacrylate and sealed methacrylate composites but not on Silorane composite. Polishing following the bleaching on the sealed composite yielded a hardness value similar to that of unsealed methacrylate composite (control).

## Introduction

Increasing trend to have more esthetic and whiter teeth has resulted in widespread use of bleaching agents in dentistry due to their availability and safety. For many years, this approach has been considered as the most conservative and cost-effective treatment method of brightening the shade of the teeth and improving the esthetic smile [1-2].

The main effective component in bleaching agents is generally two forms of peroxide agents, carbamide and hydrogen peroxide. The agents are used in at-home or in-office bleaching. In the former procedure, low concentrations of hydrogen peroxide (3%-14%) or carbamide peroxide (10%-45%) are exploited with slow effect whilst in the latter procedure; high concentrations of them with rapid effect are employed [2-4]. In the carbamide peroxide products, one third hydrogen peroxide and two thirds urea are released by process of decomposition of the products [3, 5]. The lightening effect of hydrogen peroxide is related to the high oxidative ability of the perhydroxyl free radicals created by breaking down of active hydrogen peroxide. This radical may be responsible for decomposition of pigmented macromolecules into smaller, less pigmented ones [1, 5]. 

In recent years, the higher concentrations of hydrogen peroxide used for bleaching procedures have been more attractive due to the greater oxidation power. On the other hand, the combination of in-office and at-home bleaching was recommended to yield a more effective whitening result in a lesser time [6-7]. The other advantage of this combination can be that at-home bleaching subsequent to in-office bleaching can prevent the remarkable reversal of whitening effect which happens commonly within the first two weeks of bleaching [8].

The relatively high diffusion rate of hydrogen peroxide through the enamel and dentin during bleaching time [9] may be accompanied by some deleterious side effects such as those on the existing composite restorations on the teeth involved in the bleaching process in the invisible area. The oxidation reaction may lead to chemical softening of the organic matrix of composite resins. The literature about the effects of bleaching agents on surface hardness of composite materials demonstrated dissimilar results [3-4,10-16], depending on the composition of the composite, concentration and type of bleaching agents, exposure time, and frequency of bleaching agent change [14, 17-18]. Recently, a novel resin composite, Silorane based-composite, has been introduced by Wernmann et al. [19] as a low shrinkage resin containing siloxane and oxirane functional moieties with sufficient mechanical properties, increased hydrophobicity, decreased water sorption, solubility and diffusion coefficient, resulting in high chemical stability [19-21]. These beneficial properties can enhance the longevity of the composite restorations [21]. 

In an attempt to increase the longevity of restorations, the use of surface sealants has been recommended. This resin coating layer can improve the surface roughness, wear resistance and marginal integrity of restorations [22-23]. In two recent studies, the applications of a surface sealant could minimize the adverse effect of acidic solutions on the surface hardness of composite resin [24] or bleaching agent on the marginal integrity of resin modified glass ionomer restorations [25]. In the latter study, the sealant covered the whole surface of the restoration up to 1 mm beyond its margins.

Therefore, this study evaluated the effect of the strong bleaching agent used in a combined bleaching regimen on the surface microhardness of a Silorane-based composite compared to a methacrylate-based composite. Also, the effect of a surface sealant on the latter composite was assessed when using the combined bleaching. 

## Materials and Method

Two types of resin composite, a Silorane-based microhybrid (Filtek Silorane; 3M ESPE # N/75795) and a methacrylate-based nanohybrid (Ice enamel; SDI, Bayswater, Victoria, Australia # 100761T) were used in this study. The A2 was selected as the shade of both composites. The disc-shaped specimens, 5 mm in diameter and 2 mm in depth, were prepared by packing the composites in a metallic mold between two polyester strips and thin glass plates. The plate on the top surface of the composite was gently pressed to remove the excess and voids. The specimens were cured with a light-curing unit (VIP Junior; Bisco, Schaumburg, IL, USA) at 600 mW/cm^2^ intensity according to the respective manufacturers’ curing time, for 20 seconds for the methacrylate composite and 40 seconds for the Silorane one. After complete polymerization during 24 hours storage at 37^◦^C, the top surfaces of the specimens were polished by the same operator with a medium, fine and superfine discs (Sof-Lex; 3M ESPE, St Paul, MN, USA) in a slow speed handpiece. 

The total sixty three specimens including 45 specimens from methacrylate composite and 18 specimens from Silorane composite were stored in 37^◦^C water for eight weeks to simulate the existing restorations prior to bleaching in the clinical condition. 

After the in vitro aging, 45 specimens of the Ice composite and 18 specimens of the Silorane composite were randomly divided into five groups (1-5) and two groups (6-7), respectively. 

Group 1 (methacrylate control): The specimens were kept in the storage media. 

Group 2 (sealed-methacrylate control): The top surface of the Ice composite was slightly roughened with a fine diamond bur, material dust and residue were sprayed away with water, and it was air dried. Then a thin layer of a nanofilled surface sealant (Easy Glaze; Voco GmbH, Caxhaven, Germany # 1121430) was applied on the prepared surface with the enclosed brush and light-cured for 30 seconds according to the manu-facturer’s instruction. 

Group 3 (bleached-methacrylate): The specimens were subjected to three 15-minute applications of a power bleaching gel, hydrogen peroxide 40% (Opalescence Boost; Ultradent, South Jordan, UT, USA) according to the manufacturer’s instructions; the bleaching process was then followed by an at-home bleaching gel containing carbamide peroxide 20% (Opalescence PF, Ultradent Inc, South Jordan, UT, USA) for seven days, six hours daily. 

Group 4 (sealed-bleached): First the methacrylate composite surface was covered by the sealant similar to group 2 and bleached with the same procedure employed in group 3. 

Group 5 (sealed-bleached polished): The surface covering and subsequent bleaching of the methacrylate composite surface were performed similar to the previous group (4). Then, the treated surface was polished with fine and superfine Sof-Lex (3M ESPE, USA) discs to remove the resin sealant carefully. The surfaces were examined under stereomicroscope (Carl Ziess Inc, Oberkochen, Germany) to ensure complete removal of the sealant. 

Group 6 (Silorane control): The Silorane composite specimens were not exposed to any bleaching and remained in the storage media. 

Group 7 (Silorane bleached): The specimen surfaces were bleached with the same bleaching regimen used in group 3. 

During the bleaching process, all the specimens were kept in water at 37^◦^C. In addition, tooth brushing on the specimen surfaces was done two times daily to simulate in vivo situation. 

After drying the specimens, the microhardness test was performed with a Vickers hardness testing machine (Wolpert; Darmstadt, Germany) with a 500 g load applied through the indenter and a loading time of 15 seconds. Three microharness measurements were obtained from three random positions on the surface of each specimen and a mean value was recorded as Vickers hardness number (VHN). The collected data were statistically analyzed using one-way ANOVA and Tukey tests with a significance level of 0.05.

The negative effect of the combined bleaching regimen on surface hardness of Ice composite had been verified in our previous pilot study.

## Results

Table 1 shows the mean VHN for the seven tested groups. 

**Table 1:** Microhardness values of the seven groups tested in this study

**Group**	**Group Definitions**	Mean (SD)*
1	Methacrylate control	52.16(2.30)^A^
2	Sealed methacrylate control	40.67(1.55)^C^
3	Methacrylate bleached	47.90(1.93)^B^
4	Sealed bleached	34.43(2.30)^D^
5	Sealed bleached polished	51.26(1.69)^A^
6	Silorane control	54.31(1.33)^A^
7	Silorane bleached	53.15(1.82)^A^

*The different capital letters indicate statistically significant difference (*p*<0.05)

According to results of ANOVA, there was a significant difference among the seven groups (*p*< 0.001). Group 2 (sealed-methacrylate) had a significantly lower VHN than group 1 (control) (*p*< 0.001). There was no significant difference between control groups, methacrylate and Silorane composites (*p*> 0.05). The bleaching regimen resulted in a significant lowered VHN for groups 3 and 4 (sealed and unsealed methacrylate) (*p*≤ 0.001) while it had no effect on group 6 (Silorane) (*p*> 0.05). Polishing, following the bleaching of the sealed composite (group 5), revealed a comparable VHN to group 1 (unbleached methacrylate). 

## Discussion

The preservation of surface properties of the restorative material such as surface roughness and hardness, and wear resistance besides the integrity of the material/tooth mainly determines the restoration durability [26]. Among these virtues, surface hardness as a mechanical property is directly related to the wear resistance of the material [27]. The occurrence of surface softening by bleaching agents in clinical situation can lead to wearing process of the resin. 

According to the results obtained in this study, among the unbleached composites, Silorane composite showed a similar hardness compared to methacrylate composite. Both composites had significantly higher hardness compared to the sealed composite. This difference might be attributed to the different compositions of the three material surfaces. The type, chemistry, morphology, and size of the filler have been reported to affect the material surface hardness [27-28]. The low surface hardness value of the sealed composite may be attributed to the high resin monomer content of the sealant. Similar findings were reported by recent studies when using the composite sealed with Biscover LV sealant (Bisco Inc.; USA) [24, 29]. 

There is a controversy regarding the surface hardness of the Silorane composite. One study indicated the lower hardness of Silorane composite compared to methacrylate one [30] whilst other authors have reported comparable results [31-32]. Nevertheless, our results showed approximately similar hardness for Silorane composite compared to Ice, a nanohybrid methacrylate composite. Although the Silorane composite contains 5% lower filler than that of Ice (manufactures data), the highly cross-linked polymer matrix originating from the multifunctional Silorane monomer and its hydrolytic stability may account for the comparable hardness value obtained [21, 32-33] since the hardness of unbleached composites were measured after the period of aging. The surface hardness stability of Silorane composite after food-simulating liquids or water immersion was previously reported [34-35]. 

In the current study, microhardness of Silorane composite was not altered after bleaching procedures. This result was expected based on the high chemical stability and hydrophobicity of Silorane matrix [20-21, 32]. On the contrary, the bleaching agents significantly decreased the microhardness of the sealed (in particular) and unsealed methacrylate composites. The possible explanation for this finding may be that the hydrogen peroxide had a softening effect on the resin matrix [4, 11]. Following bleaching, oxidation reaction can occur in polymer chain of the resin matrix; which is responsible for a more reduced microhardness for the material containing greater resin matrix [11]. The chemical degradation of the resin matrix was reported when using the concentrated or repeated application of hydrogen peroxide [36]. The free radicals, released by breaking down of hydrogen peroxide, finally combine to form oxygen molecule and water [19]. Some factors involved in this chemical process might accelerate the hydrolytic degradation of the composites demonstrated by Soderholm and others [37]. The adverse effect of hydrogen peroxide on the filler- resin interface may result in filler-matrix debonding by water uptake [18]. Subsequently, displacement of the filler particles can occur, as observed in a SEM study. This phenomenon led to a decreased microhardness of the nanocomposite Premise (Kerr;USA) after at-home bleaching [38].

The high peroxide concentrations used in the current study may facilitate the cumulative softening effects of the whitening agents, in-office and at-home agents. The importance of peroxide concentration and pH of bleaching agents in having the adverse effects on restorations has been noticed [18, 39-40]. The greater hydrogen peroxide release from higher concentration of the carbamide peroxide gel and a resultant lowered microhardness value was reported for the Charisma (Heraeus; Kulzer, Germany) and Vitremer (3M ESPE, USA) [39]. The same effect was demonstrated for Spectrum TPH (Dentsply; USA) and Fuji II LC (GC; Tokyo, Japan) treated with Opalescence Xtra (Ultradent; USA, 35% hydrogen peroxide) compared to Opalescence Quick (Ultradent; USA, 35% carbamide peroxide). The former had a low pH (3.67) and the latter had a high pH (6.53) [14]. However, in that study [14], no significant difference was reported between the control and bleached groups for the tested materials. The pH of most bleaching products is approximately neutral. The pH of Opalescence Boost after mixing and Opalescence PF used in the present study was 6.6-7.6 and 6.5, respectively. However, a rise in the pH value of Opalescence PF used in the present can occur following decomposition of carbamide peroxide into hydrogen peroxide and urea because urea decomposes into CO_2_ and a strong base, ammonia. This higher pH is capable of producing more perhydroxyl-free radicals [41]. Lack of any changes in microhardness of different esthetic restorative materials after bleaching was observed in previous studies [10, 12, 14, 17, 39, 42-43]. Some studies demonstrated a positive effect [10, 15-16, 38] or a decreasing effect similar to our result on the surface hardness [1, 11, 16, 38, 44]. As mentioned earlier, difference in compositions of the restorative materials and bleaching products, various pH value of bleaching agents, testing methodology and different simulating clinical conditions could contribute to the conflicting reported results. 

In the present study, although the use of the bleaching regimen resulted in a decreased hardness of sealed composite, accurate polishing could re-establish the original microhardness value, thereby has a clinical benefit. Therefore, the formed uniform resin coating might act as a protective layer which prevented the contact between the bleaching agent and composite surface. The softening effect of hydrogen peroxide may be limited to this protective layer and removed by polishing. Furthermore, according to Yu et al. [25]; possible extension of the resin layer on the enamel surface and subsequent polishing did not alter the favorable bleaching outcome. 

The softening effect of the carbamide peroxide restricted to the surface layer of composite restorations was supported by Lima et al. [44]. These authors suggested re-polishing of the softened composite surface after bleaching with 16% carbamide peroxide. More et al. [45] recommended to polish composite restoration after bleaching to eliminate the roughened outer surface, preventing the adherence of the microorganisms. However, Hanning et al. study [46] demonstrated a decreased microhardness of deep layers in the resin restorations treated by different bleaching techniques. They concluded that polishing may not be able to re-establish the surface hardness of the filling after the bleaching [46].

Further studies are needed to confirm our results and to assess these experimental conditions for different brands of composite materials. Furthermore, other factors may influence the results in clinical situation. Water or saliva may dilute or buffer the bleaching agents. Formation of a protective salivary layer, particularly its protein content, might modify or decrease the effects of bleaching agents on the restoration in the intraoral situation [4, 25]. On the other hand, it was reported that absorption of salivary proteins by the composite surface decreased after bleaching [47]. Therefore, the impact of bleaching procedures on the existing restorations of different patients can be influenced by a complex phenomenon.

## Conclusion

Considering the limitation of this *in vitro* study, it can be concluded that the combined bleaching with a high concentration of hydrogen peroxide exhibited no effect on Silorane composite while it had an adverse effect on the sealed and unsealed methacrylate composite in terms of microhardness. The prior sealing, then bleaching and followed polishing would re-establish the surface hardness of the methacrylate composite. Therefore, it is safe to suggest that using a surface sealant on restorative materials prior to bleaching presents a preservative function against any adverse effect of the bleaching agents.
